# PLK4: Master Regulator of Centriole Duplication and Its Therapeutic Potential

**DOI:** 10.1002/cm.22031

**Published:** 2025-04-21

**Authors:** Muhammad Hamzah, Franz Meitinger, Midori Ohta

**Affiliations:** ^1^ Okinawa Institute of Science and Technology Graduate University Okinawa Japan

**Keywords:** cancer, centrioles, centrosome, kinase inhibitor, mitosis, pericentriolar material, therapeutics

## Abstract

Centrosomes catalyze the assembly of a microtubule‐based bipolar spindle, essential for the precise chromosome segregation during cell division. At the center of this process lies Polo‐Like Kinase 4 (PLK4), the master regulator that controls the duplication of the centriolar core to ensure the correct balance of two centrosomes per dividing cell. Disruptions in centrosome number or function can lead to genetic disorders such as primary microcephaly or drive tumorigenesis via centrosome amplification. In this context, several chemical inhibitors of PLK4 have emerged as promising therapeutic candidates. The inhibition of PLK4 results in the emergence of acentrosomal cells, which undergo prolonged and error‐prone mitosis. This aberrant mitotic duration triggers a “mitotic stopwatch” mechanism that activates the tumor suppressor p53, halting cellular proliferation. However, in a multitude of cancers, the efficacy of this mitotic surveillance mechanism is compromised by mutations that incapacitate p53. Recent investigations have unveiled p53‐independent vulnerabilities in cancers characterized by chromosomal gain or amplification of 17q23, which encodes for the ubiquitin ligase TRIM37, in response to PLK4 inhibition, particularly in neuroblastoma and breast cancer. This review encapsulates the latest advancements in our understanding of centriole duplication and acentrosomal cell division in the context of TRIM37 amplification, positioning PLK4 as a compelling target for innovative cancer therapeutics.

## 1 Introduction

**FIGURE 1:**
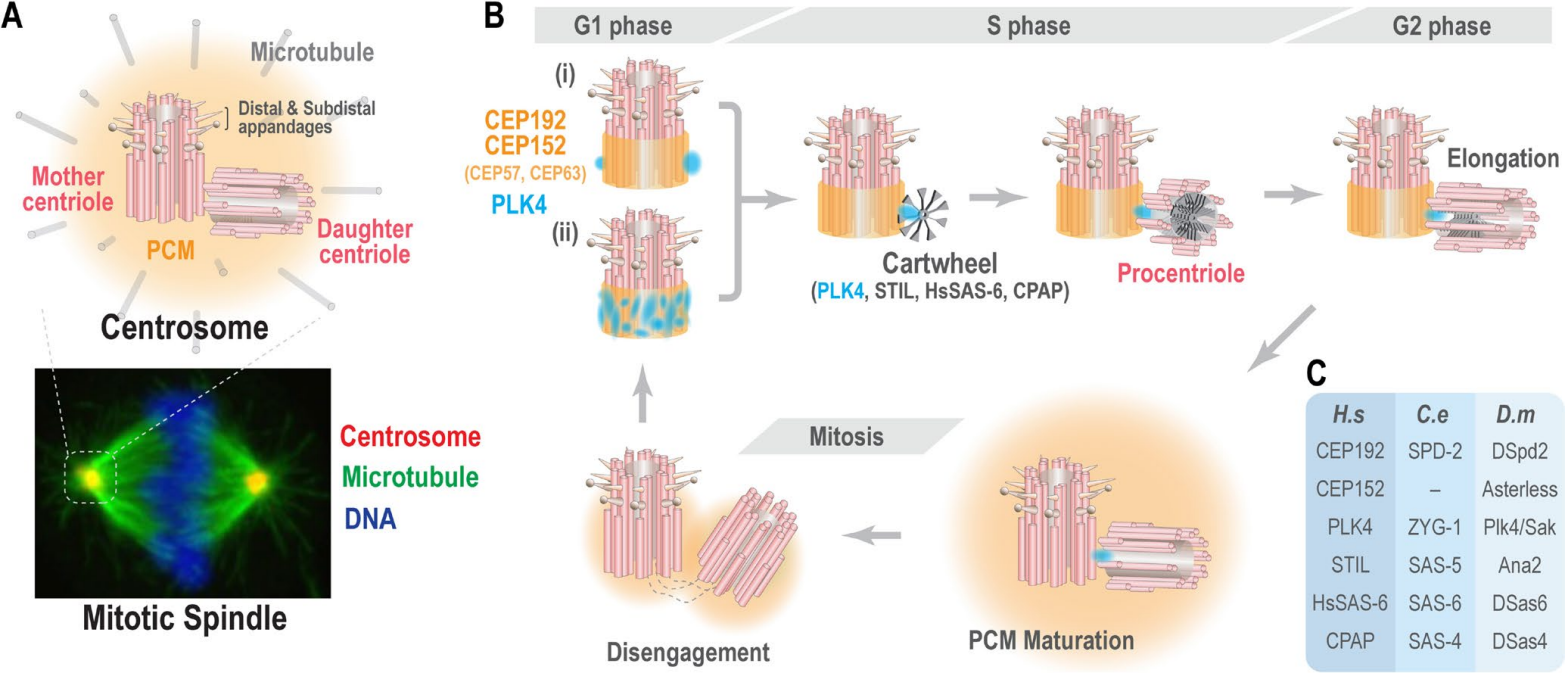
Centrosome duplication cycle. (A) Schematic of the centrosome, showing centrioles surrounded by the pericentriolar material (PCM), essential for microtubule nucleation and centrosome function. (B) Stepwise depiction of daughter centriole assembly and centrosome maturation across cell cycle phases, highlighting critical transitions. (i) and (ii) highlight two recent observations of PLK4 distribution before centriole duplication. (C) List of conserved genes across species— *Homo sapiens* (*H.s*), *Drosophila melanogaster* (*D.m*), and *Caenorhabditis elegans* (*C.e*)—required for PLK4‐driven daughter centriole formation, illustrating the evolutionary conservation of centriole biogenesis.

Centrosomes serve as the primary microtubule‐organizing centers (MTOCs) in metazoans, catalyzing spindle assembly to ensure proper chromosome segregation (Figure 1A) (Nigg and Holland 2018). Centrosomes are composed of cylindrical centriolar cores surrounded by a proteinaceous matrix known as the pericentriolar material (PCM). During mitosis, the PCM increases in size and microtubule nucleating capacity by recruiting microtubule nucleating factors such as γ‐tubulin ring complexes (γ‐TuRC) and mitotic kinases. To ensure that a cell in mitosis has precisely two centrosomes that facilitate bipolar spindle assembly, the centriolar core duplicates once per cell cycle (Figure 1B). This duplication process is tightly regulated by Polo‐Like Kinase 4 (PLK4) in humans (Habedanck et al. 2005; Kleylein‐Sohn et al. 2007), Plk4/Sak in *Drosophila melanogaster* (Bettencourt‐Dias et al. 2005), and ZYG‐1 in *Caenorhabditis elegans* (O'Connell et al. 2001) (Figure 1C).

Overexpression of PLK4 promotes centriole overduplication and *de novo* centriole formation (Habedanck et al. 2005; Peel et al. 2007; Rodrigues‐Martins et al. 2007), leading to multipolar spindle formation and chromosome instability (Ganem et al. 2009). Inhibition of PLK4 inversely leads to loss of centriole assembly, resulting in monopolar spindle formation and centrosome loss (Bettencourt‐Dias et al. 2005; Habedanck et al. 2005; O'Connell et al. 2001; Wong et al. 2015). Consequently, the expression and activation of PLK4 are meticulously regulated in normal cells to ensure accurate chromosome segregation. Recent advancements in imaging, structural biology, and gene editing have shed light on the mechanisms that control PLK4 activity, ensuring centriole duplication occurs once per cell cycle. In addition, targeted inhibition of PLK4 has emerged as a promising therapeutic strategy in the fight against cancer, supported by recent preclinical studies investigating the synthetic‐lethal relationship between TRIM37 amplification and PLK4 inhibition (Meitinger et al. 2020; Therapeutics 2023; Yeow et al. 2020). This review highlights key concepts of PLK4‐mediated centriole duplication and emphasizes the underlying mechanisms of cancer‐specific susceptibility to PLK4 inhibition.

## 2 Centriole Duplication

Centrosomes contain a pair of centrioles, each formed in different cell cycles. The matured centriole, called the mother centriole, is decorated with distal and sub‐distal appendages, while the newer centriole, known as the daughter centriole, lacks appendages and is generated in the previous cell cycle (Figure 1A,B). The distal and sub‐distal appendages function as a membrane docking platform in the initial phase of ciliogenesis and a signaling hub for activating the PIDDosome pathway to protect cells from centriole amplification (Breslow and Holland 2019; Burigotto et al. 2021; Evans et al. 2021; Fava et al. 2017). Centrioles have a conserved 9‐fold symmetric structure composed of stabilized microtubules. In most cases, including vertebrates and *Chlamydomonas*, the microtubule blades are triplets (Geimer and Melkonian 2004; Gonczy 2012; Guichard et al. 2010; Paintrand et al. 1992), whereas doublets are observed in *D. melanogaster* embryos and somatic cells, and singlets in *C. elegans* embryos (Gonzalez et al. 1998; Gottardo et al. 2015; Pelletier et al. 2006; Sugioka et al. 2017). A recent cryo‐electron tomography study in *C. elegans* embryos has revealed variations in microtubule arrangements with centriole maturation: mother centrioles contain doublets, while daughter centrioles have singlets (Tollervey et al. 2024). The mechanism by which daughter centrioles acquire doublet microtubules during maturation remains unclear.

In late G1 to early S phase, centrioles begin to duplicate, with new daughter centrioles (procentrioles) assembling perpendicularly and adjacent to the mother centrioles (Figure 1B). The initial duplication involves two steps. First, key components of the precursor structure, known as the cartwheel, are recruited to the mother centrioles. Second, these components assemble into the cartwheel structure, providing a scaffold for centriole duplication (Dammermann et al. 2008; Dzhindzhev et al. 2017; Dzhindzhev et al. 2014; Gonczy 2012). The cartwheel is the first structure with 9‐fold symmetry, where each unit consists of a central hub and nine spokes that extend radially to the microtubule blades. These units stack within the lumen to create a three‐dimensional cartwheel structure (Guichard et al. 2012; Guichard et al. 2013). Initially, centrioles in *C. elegans* embryos were thought to lack a cartwheel, as electron microscopy (EM) revealed only a simple central tube within the lumen (Pelletier et al. 2006). However, recent studies using Ultrastructure Expansion (U‐Ex) microscopy with STimulated Emission Depletion (STED) microscopy or cryo‐tomography have shown that *C. elegans* centrioles indeed possess a cartwheel‐like structure at their core (Tollervey et al. 2024; Woglar et al. 2022). Following cartwheel formation, the microtubule blades assemble and elongate to form the daughter centrioles. As cells enter mitosis, the PCM matrix expands, nucleating spindle microtubules for chromosome segregation. Additionally, mother and daughter centrioles disengage through cleavage of the PCM, preparing them for the next cycle of centriole duplication (Tsou and Stearns 2006).

## 3 Spatiotemporal Dynamics of PLK4 in Centriole Duplication

Pioneering genetic screens in *C. elegans* have identified core proteins essential for centriole duplication, including the kinase ZYG‐1 (homologous to PLK4 in humans and Plk4/Sak in *Drosophila*), SPD‐2 (Cep192/DSpd2), SAS‐4 (CPAP/DSas4), SAS‐5 (STIL/Ana2), and SAS‐6 (HsSAS‐6/DSas6) (Dammermann et al. 2004; Delattre et al. 2004; Kemp et al. 2004; Kirkham et al. 2003; Leidel et al. 2005; Leidel and Gonczy 2003; O'Connell et al. 2001; Pelletier et al. 2004) (Figure 1C). Over the past two decades, research employing diverse models, including *Drosophila* and human cell cultures, has identified orthologs of these components and highlighted their conserved roles in centriole formation across eukaryotic evolution (Andersen et al. 2003; Arquint et al. 2012; Basto et al. 2006; Bettencourt‐Dias et al. 2005; Dix and Raff 2007; Habedanck et al. 2005; Hung et al. 2000; Kitagawa et al. 2011a; Kleylein‐Sohn et al. 2007; Peel et al. 2007; Pelletier et al. 2006; Stevens et al. 2010; Tang et al. 2011; Vulprecht et al. 2012; Zhu et al. 2008). In this review, human protein names are mainly used, except when discussing organism‐specific topics.

PLK4 is the master regulator of centriole duplication (Figure 1B) (Bettencourt‐Dias et al. 2005; Habedanck et al. 2005; O'Connell et al. 2001). During the G1 phase, human CEP192 and CEP152 localize around the mother centriole with the assistance of CEP57 and CEP63 and recruit PLK4 through direct binding (Figure 1B) (Brown et al. 2013; Cizmecioglu et al. 2010; Dzhindzhev et al. 2010; Hatch et al. 2010; Kim et al. 2013; Kim et al. 2019; Lukinavicius et al. 2013; Sir et al. 2011; Sonnen et al. 2013; Wei et al. 2020). Asterless (*Drosophila* ortholog of CEP152) was identified as the crucial PLK4 receptor in *Drosophila*, while DSpd2 (*Drosophila* ortholog of CEP192) is essential for PCM maturation but not for PLK4 recruitment (Dix and Raff 2007; Dzhindzhev et al. 2010). Interestingly, *C. elegans* lacks a CEP152 ortholog, and instead, SPD‐2 ( *C. elegans* ortholog of CEP192) directly recruits ZYG‐1 to centrioles (Figure 1C) (Delattre et al. 2006; Pelletier et al. 2006). By combining sample expansion, STED imaging, and electron microscopy, recent work from Sullenberger and colleagues has built a precise distribution map of the PLK4 scaffold proteins, proposing CEP152 as the primary receptor for PLK4 in humans (Sullenberger et al. 2023). This highlights an intriguing evolutionary divergence in the scaffold molecules responsible for PLK4 recruitment, raising questions about how these mechanisms have evolved to initiate centriole duplication across species.

Before centriole duplication, PLK4 localizes at the proximal end of the mother centriole in the G1 phase (Figure 1Bi,ii). Earlier studies in cultured human cells using resolution‐limited imaging and structured illumination microscopy have described this PLK4 localization as ring‐like (Kim et al. 2013; Ohta et al. 2014). Recent advancements in STED microscopy and expansion microscopy have led to two distinct observations of PLK4 localization in the G1 phase. Scott and colleagues reported that PLK4 localized as a single spot or as multiple distinct foci encircling mother centrioles (Scott et al. 2023) (Figure 1Bi). In contrast, Sullenberger et al. showed that PLK4 occupies a cylindrical arrangement around mother centrioles, as previously reported (Sullenberger et al. 2023) (Figure 1Bii). They noted that PLK4 signals could appear in isolation, surrounded by signal‐less areas, rather than forming a continuous ring. These distinct observations may result from the transient assembly and instability of PLK4 cylindrical distribution. Further investigations will be required to clarify this discrepancy. As cells enter the S phase, PLK4 binds directly to STIL (SCL/TAL1 interrupting locus) and recruits it to the site of centriole duplication (Figure 1B) (Arquint et al. 2015; Dzhindzhev et al. 2014; Kratz et al. 2015; Ohta et al. 2014). This interaction activates PLK4 and stabilizes it at the duplication site as a single dot while removing it from the surrounding regions (Moyer et al. 2015; Ohta et al. 2014; Ohta et al. 2018). The restriction of PLK4 to a single site is thought to ensure that each mother centriole produces only one procentriole. Several mathematical models have been proposed to describe this transition in PLK4 localization patterns (Leda et al. 2018; Takao et al. 2019; Wilmott et al. 2023).

**FIGURE 2:**
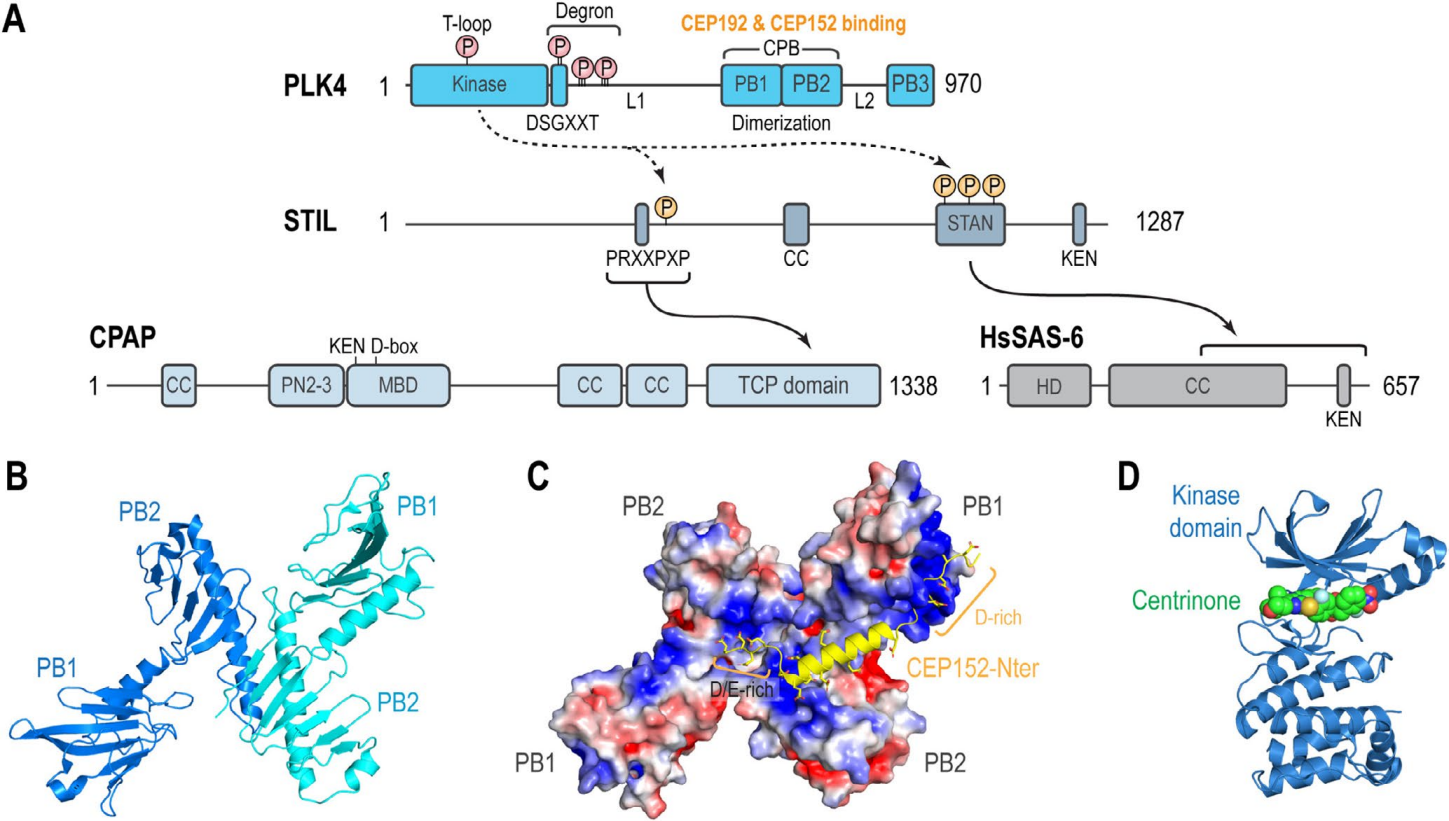
PLK4 initiates molecular interactions of centriole duplication factors. (A) Schematic representation of PLK4, STIL, CPAP, and HsSAS‐6, highlighting key structural domains, interactions, and phosphorylation sites involved in the regulation of daughter centriole formation. Depicted are the interactions (arrows) between PLK4 and essential substrates (broken arrows) that facilitate centriole biogenesis. The trans‐autophosphorylation sites in PLK4 are colored red, while PLK4 phosphorylation sites in STIL are colored yellow. MBD: microtubule‐binding domain. (B) Structural illustration of the cryptic polo box (CPB) domain modified from PDB:4N7V. (C) Electrostatic surface representation of the CPB‐CEP152 N‐terminus binding, modified from PDB: 4N7V, which is critical for the recruitment of PLK4 to centrioles before daughter centriole formation. (D) Kinase domain of PLK4 binding to Centrinone, modified from PDB:4YUR.

The restriction of PLK4 to a single site is primarily controlled by its trans‐autophosphorylation, which removes excess PLK4 around mother centrioles (Figures 1B and 2A) (Dzhindzhev et al. 2017; Ohta et al. 2018; Scott et al. 2023; Yamamoto and Kitagawa 2019). PLK4 homodimers increase their kinase activity by trans‐autophosphorylating threonine 170 (in humans) or 172 (in *Drosophila*) within the activation loop (T‐loop) of its kinase domain (Klebba et al. 2015a; Lopes et al. 2015; Moyer et al. 2015; Nakamura et al. 2013). Additionally, they trans‐autophosphorylate a degron, creating a binding site for the SCF‐Slimb/βTrCP‐E3 ubiquitin ligase, which subsequently targets PLK4 for ubiquitination and proteasomal degradation (Figure 2A) (Cunha‐Ferreira et al. 2013; Cunha‐Ferreira et al. 2009; Guderian et al. 2010; Holland et al. 2010; Klebba et al. 2013; Rogers et al. 2009; Sillibourne et al. 2010). This trans‐autophosphorylation is counteracted by protein phosphatases (PP) such as PP1, PP2A/Twins, and PP5, which interact with PLK4/ZYG‐1 and regulate its stability and interaction partners (Abraham et al. 2023; Brownlee et al. 2011; Peel et al. 2017). Previous FRAP assays in cells showed that the centriolar turnover of overexpressed PLK4 depends on its kinase activity and trans‐autophosphorylation (Yamamoto and Kitagawa 2019). More recent FRAP analysis in cells with endogenously fluorescent‐tagged PLK4 demonstrated the centriolar turnover of PLK4 increases during the S phase compared to the G1 phase, suggesting higher PLK4 activity in the S phase (Scott et al. 2023). Furthermore, CEP85, which binds and recruits STIL to centrioles, may collaborate with STIL to activate PLK4 (Liu et al. 2018). In cultured *Drosophila* cells, PLK4 is briefly detected as a ring‐like distribution during early mitosis, which is rapidly resolved into a single focus during mitotic exit when centriole duplication begins (Aydogan et al. 2020; Dzhindzhev et al. 2017). Along with Ana2 (the *Drosophila* ortholog of STIL) and Asterless (the *Drosophila* ortholog of CEP152), which bind and activate PLK4 to initiate centriole duplication, the local concentration of PLK4 is also sufficient to activate centriole duplication independently (Boese et al. 2018; Dzhindzhev et al. 2017; Dzhindzhev et al. 2014; Klebba et al. 2015a; Klebba et al. 2015b; Lopes et al. 2015; McLamarrah et al. 2018; Ryniawec et al. 2023). Structural insights into these interactions are needed to fully understand how PLK4 is simultaneously activated and stabilized at the centriole duplication site through binding to the activators.

## 4 PLK4 Substrates Initiate Cartwheel Formation

At the onset of centriole duplication in human cells, PLK4 phosphorylates the conserved C‐terminal STAN domain of STIL, facilitating its interaction with HsSAS‐6, a key component essential for assembling the 9‐fold symmetric cartwheel structure (Figure 2A) (Arquint et al. 2015; Kitagawa et al. 2011b; Kratz et al. 2015; Moyer et al. 2015; Nakazawa et al. 2007; Ohta et al. 2014; van Breugel et al. 2011). PLK4 also phosphorylates the N‐terminal domain of STIL, promoting interaction with CPAP, which is crucial for forming and stabilizing the triplet microtubule blades that constitute the procentriole wall (Moyer and Holland 2019). These phosphorylation‐dependent interactions are conserved in *Drosophila*, where Ana2 links the growing cartwheel to the microtubule wall (Dzhindzhev et al. 2014; McLamarrah et al. 2020). In *C. elegans* , however, SAS‐5 ( *C. elegans* ortholog of STIL) interacts directly with SAS‐6 without phosphorylation (Kitagawa et al. 2009; Leidel et al. 2005; Lettman et al. 2013). Recent findings revealed that ZYG‐1 phosphorylates the N‐terminus of SAS‐5 to stabilize cartwheel assembly and the C‐terminus to prevent excess centriole formation (Sankaralingam et al. 2024).

Recent advances in U‐Ex microscopy have mapped the spatial organization of 24 centriolar proteins and generated a time‐series reconstruction of their distribution during human procentriole assembly (Laporte et al. 2024). This analysis revealed that PLK4, HsSAS‐6, STIL, and CPAP localize to the naked cartwheel layer in the earliest stages of centriole biogenesis, preceding microtubule blade assembly. Critical questions remain regarding the stoichiometry and assembly mechanisms of these cartwheel components and how PLK4 forms stable complexes while avoiding trans‐autophosphorylation‐mediated degradation.

## 5 PLK4 Structure and Inhibitors

PLK4 is a member of the Polo‐Like Kinase (PLK) family, which is best known for its regulatory roles in cell cycle events, including the DNA damage response and cell division (Archambault and Glover 2009; Zitouni et al. 2014). PLK family members share a highly conserved N‐terminal serine/threonine kinase domain and a C‐terminal region containing one or more polo boxes (PB) (Barr et al. 2004). In most PLKs, two conserved PBs combine to form a single PB domain crucial for subcellular targeting and kinase regulation (Elia et al. 2003). However, PLK4 is different: it has only one PB (PB3) at its outermost C‐terminus (Fode et al. 1994; Leung et al. 2002) and a central, divergent “cryptic polo box” (CPB) that forms a homodimer for target binding and subcellular localization (Figure 2A) (Habedanck et al. 2005; Leung et al. 2002; Slevin et al. 2012; Swallow et al. 2005).

The CPB consists of two tandem polo boxes, PB1 and PB2 (Slevin et al. 2012), which form an antiparallel dimer through a β‐sheet in PB2 (Figure 2B) (Park et al. 2014; Shimanovskaya et al. 2014). This dimerization creates a basic patch on the CPB surface, facilitating interactions with acidic regions of receptor proteins such as CEP192 and CEP152 in humans, SPD‐2 in *C. elegans* , and Asterless in *Drosophila* (Figure 2C) (Park et al. 2014; Shimanovskaya et al. 2014). In contrast to PLK1's PB domain which exclusively binds to phosphorylated peptides (Elia et al. 2003; Yun et al. 2009), PLK4's CPB interacts with unphosphorylated substrates (Park et al. 2014; Shimanovskaya et al. 2014). Additionally, the interaction between the PB3 domain of PLK4 and the coiled‐coil region of STIL is required for initiating centriole duplication (Arquint et al. 2015). Furthermore, PLK4's Linker 1 (L1) domain, located between the kinase and CPB domains, also contributes to STIL binding (Arquint et al. 2015; Ohta et al. 2018). Understanding the coordination of these multidomain interactions is crucial for elucidating how PLK4 activity and stability are regulated to ensure the formation of a single daughter centriole per cell cycle.

PLK4 has a highly conserved Gly‐X‐Gly‐X‐X‐Ala motif within the kinase domain, which is crucial for adenosine triphosphate (ATP) binding and phosphate transfer to the substrate (Fode et al. 1994; Golsteyn et al. 1996; Hanks et al. 1988). Targeting this motif in PLK4 with small molecules has been considered a therapeutic strategy for blocking centriole duplication in cancers that rely on PLK4‐driven centrosome amplification, which is frequently observed in certain cancers, particularly breast cancer and melanoma (Figure 2D) (Chan 2011; Denu et al. 2018; Denu et al. 2016; Zhao and Wang 2019). To date, several PLK4 inhibitors, including CFI‐400945, Centrinone, Centrinone B, YLT‐11, and YLZ‐F5, have been characterized in cancer cell lines and in vivo xenograft models (Table 1) (Lei et al. 2018; Mason et al. 2014; Wong et al. 2015; Zhu et al. 2020). An orally bioavailable PLK4 inhibitor (RP‐1664) recently entered clinical trials based on promising preclinical data (Therapeutics 2023). For a comprehensive review of PLK4 inhibitors, Garvey et al. (2021) provide a detailed analysis. Here, we will focus on two widely used inhibitors in cell biology and cancer research: CFI‐400945 and Centrinone/Centrinone B (Mason et al. 2014; Wong et al. 2015).

**TABLE 1:** PLK4 inhibitors.

Name	Centrinone	Centrinone B	CFI‐400945	YLT‐11
	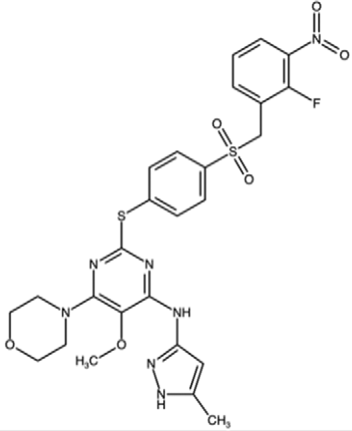	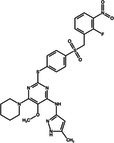	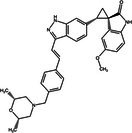	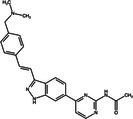
Effect on centrosomes	Centrosome depletion		Centrosome amplification (low concentration); Centrosome depletion (high concentration)	Centrosome amplification (low concentration); Centrosome depletion (high concentration)
Notable phenotypes	Prolonged mitosis; Increased PLK4 stability; Synthetic lethality to TRIM37 amplification		Cell division failure; Apoptosis; Increased PLK4 stability	Cell division failure; Apoptosis; Decreased PLK4 stability
Notable off‐target effect	None		Aurora B inhibition	Not determined
Genetic evidence for specificity	PLK4‐G95L mutation interfered with Centrinone binding; No off‐target effects were observed		Not determined	Not determined

CFI‐400945 is a PLK4 inhibitor developed by the Campbell Family Institute (CFI) that targets the ATP binding site of the kinase (Sampson et al. 2015). This orally active drug has demonstrated potent anti‐tumor activity in breast and lung cancer cells in vivo (Kawakami et al. 2018; Mason et al. 2014). Treatment with high concentrations of CFI‐400945 reduced the number of centrioles to one, whereas lower concentrations induced the formation of extra centrioles. This bimodal effect could be explained by partial inhibition of PLK4, which not only regulates centriole duplication through STIL phosphorylation but also its own degradation via trans‐autophosphorylation (Holland and Cleveland 2014). Partial inhibition can stabilize PLK4, leading to increased protein abundance and paradoxical centriole amplification. High and low doses of CFI‐400945 both eventually led to mitotic defects, resulting in cell death or arrest. Interestingly, in lung cancer cells, the drug induced the extensive formation of polyploid cells, a phenotype thought to be partly due to off‐target inhibition of Aurora kinase B (Kawakami et al. 2018; Mason et al. 2014; Oegema et al. 2018; Suri et al. 2019). This off‐target effect is likely attributable to the high similarity between the kinase domains of PLK4 and Aurora A/B kinases (Levinson 2018). Accordingly, the reported EC_50_ for PLK4 (12.3 nM) and Aurora B (102 nM) are relatively close to each other (Mason et al. 2014). CFI‐400945 has recently entered clinical trials and shows promise as an antineoplastic agent (Mason et al. 2014; Murphy et al. 2024; Suri et al. 2019; Veitch et al. 2019).

Centrinone is a reversible PLK4 inhibitor with more than 1000‐fold selectivity over Aurora A and B kinases (Wong et al. 2015). An optimized version, Centrinone B, shows even greater selectivity, with over 2000‐fold selectivity for PLK4 over Aurora A and more than 9500‐fold selectivity for PLK4 over Aurora B. Centrinone was developed using VX‐680, a pan‐Aurora kinase inhibitor that also targets PLK4, as a template. Its high specificity is achieved by introducing a methoxy substituent at the C5 position of VX‐680, which targets the unique hinge‐region methionine (Met91) in PLK4. This highly selective PLK4 inhibition effectively blocks centriole duplication over multiple cell divisions, leading to the progressive depletion of centrioles and the generation of acentrosomal daughter cells. Centrinone treatment has led to the complete elimination of centrosomes across a wide range of both transformed and non‐transformed cell lines, contributing to hundreds of studies exploring its effects (Oegema et al. 2018; Wong et al. 2015). It has become an invaluable tool for investigating acentrosomal cell cycle regulation, spindle dynamics, organelle interactions, and centriolar satellite proteins, demonstrating its versatility in studying centrosome‐related mechanisms (Takeda et al. 2020). In addition, Centrinone can be used to induce centrosome amplification (Wong et al. 2015). This seemingly paradoxical mechanism leverages the role of PLK4 as a “suicide kinase”, promoting its own degradation through kinase activity. Inhibiting PLK4's kinase activity results in the accumulation of PLK4 protein. Upon Centrinone washout, the surplus of active PLK4 triggers the formation of multiple daughter centrioles, which subsequently mature into centrosomes. This unique property of Centrinone provides a valuable tool for studying procentriole formation and centriole biogenesis (Sullenberger et al. 2023).

PLK4 inhibitors are being explored as potential therapeutic agents for cancers that depend on functional centrosomes. However, recent studies identified acentrosomal cells in ovarian and prostate tumors (Morretton et al. 2022; Wang et al. 2020). Wang and colleagues suggested that centrosome loss may enhance the oncogenic potential by increasing chromosomal instability. They demonstrated that chemical depletion of centrosomes elevated genomic instability and transformed prostate epithelial cells into tumors in mice (Wang et al. 2020). Therefore, while PLK4 inhibitors could be beneficial for treating certain cancers, they may also pose a risk of inducing secondary malignancies.

In the following sections, we summarize the latest research on PLK4 inhibition‐induced acentrosomal cell division and the associated cancer‐specific vulnerabilities.

## 6 Acentrosomal Cell Division

**FIGURE 3:**
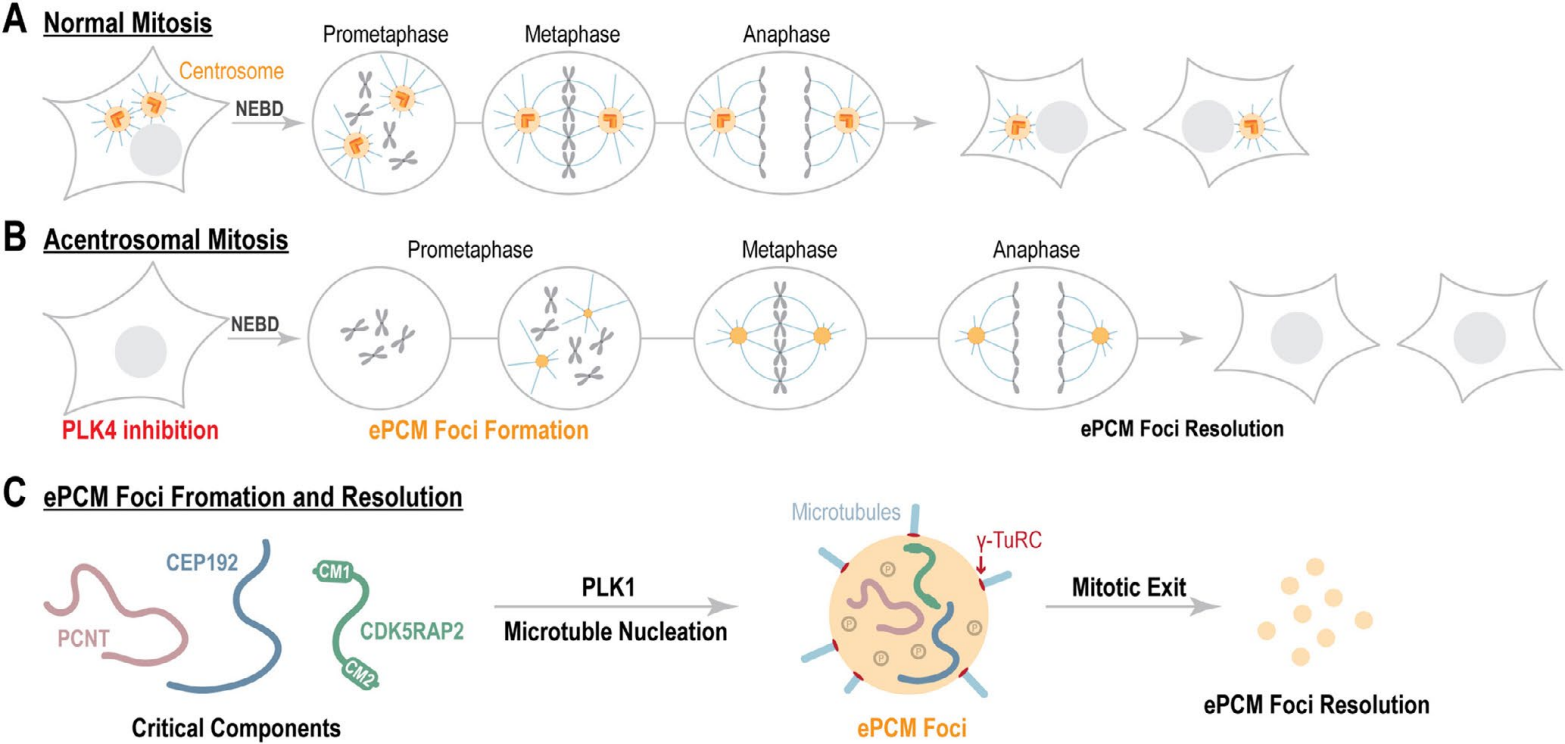
Acentrosomal cell division. (A) During normal mitosis, centrosomes facilitate microtubule nucleation, leading to the formation of a bipolar spindle that ensures accurate chromosome segregation. (B) In acentrosomal cells, ectopic pericentriolar material (ePCM) foci form, with the assistance of PLK1 and microtubules, to support bipolar spindle assembly. (C) The formation of ePCM foci depends on the coordinated action of the centrosomal proteins CDK5RAP2, PCNT, and CEP192.

In mammals, centrosomes play a crucial role in ensuring mitotic fidelity in somatic cells by promoting spindle bipolarization (Nigg and Holland 2018). In human and mouse‐derived cell lines, it was shown that centrosomes can be chemically depleted by PLK4 inhibition (Lambrus et al. 2015; Wong et al. 2015). Similarly, genetic inactivation of the essential centriolar protein SAS‐4 (CPAP) or SAS‐6 (HsSAS‐6) leads to centrosome loss in mouse embryos (Bazzi and Anderson 2014; Grzonka and Bazzi 2024). Notably, acentrosomal cells could be observed following Centrinone treatment, but not after CFI‐400945 treatment, which could be explained by Aurora B‐mediated off‐target effects that induce cytokinesis failure (Mason et al. 2014; Oegema et al. 2018). Surprisingly, somatic cells lacking functional centrosomes can still form mitotic spindles, albeit at a slower pace and with a higher risk of chromosome missegregation (Figure 3A,B) (Khodjakov and Rieder 2001; Lambrus et al. 2015; Sir et al. 2013; Wang et al. 2020; Wong et al. 2015). This raises the question: how do acentrosomal cells compensate for the loss of their primary MTOCs, which are responsible for microtubule nucleation and organization of the mitotic spindle?

Acentrosomal cell division is common in oocytes (Schuh and Ellenberg 2007; So et al. 2019), but the underlying mechanisms are fundamentally different from acentrosomal somatic cells. Several centrosome‐independent mechanisms have been identified as contributors to mitotic spindle formation in somatic cells (Kraus et al. 2023). These mechanisms include active Ran GTP, which releases spindle assembly factors near chromosomes (Halpin et al. 2011); the chromosomal passenger complex, which stabilizes chromatin‐dependent microtubules (Sampath et al. 2004); the fibrous corona, which nucleates microtubules at kinetochores (Wu et al. 2023); and the augmin complex, which is essential for microtubule branching (Goshima et al. 2008). While the augmin complex is critical even in the presence of centrosomes, the roles of kinetochore‐ and chromatin‐related microtubule nucleation mechanisms in acentrosomal cell division remain poorly understood.

So far, two mechanisms have been proposed to promote acentrosomal cell division in somatic cells. One mechanism depends on PCM matrix proteins that assemble ectopic foci to compensate for centrosome loss, and the other depends on the nuclear mitotic apparatus protein (NuMA) (Chinen et al. 2020; Chinen et al. 2021; Watanabe et al. 2020). Two independent studies showed that the PCM proteins Pericentrin (PCNT) and CDK5RAP2 (CEP215) promote bipolar spindle formation in the absence of centrioles (Chinen et al. 2021; Watanabe et al. 2020). Both studies used the PLK4 inhibitor Centrinone to deplete centrosomes. PCNT and CDK5RAP2 are dispensable for bipolar spindle formation in human cell lines (hTERT RPE1‐1, HeLa, DLD1) but are required in the absence of centrioles. It was found that during acentrosomal mitosis, PCNT, CDK5RAP2, and the essential protein CEP192 form ectopic foci that localize at the two spindle poles (Figure 3C) (Meitinger et al. 2020; Watanabe et al. 2020). Deletion of PCNT or CDK5RAP2 impairs foci formation of the other and of CEP192, suggesting that all three proteins are components of the same structure, which we name hereafter ectopic PCM (ePCM) foci. Furthermore, both deletion mutants failed to form a bipolar spindle, suggesting that the ePCM foci form centrosome‐like structures that promote bipolar spindle formation.

The ePCM foci were shown to form transiently during mitosis. They form several minutes after mitotic entry (nuclear envelope breakdown) and resolve following mitotic exit, suggesting that mitosis‐specific signals drive foci nucleation and maintenance (Watanabe et al. 2020). One such signal is the mitotic kinase PLK1, whose chemical inhibition prevented the formation of ePCM foci (Watanabe et al. 2020). Furthermore, the authors have shown that microtubule nucleation activity is required for ePCM nucleation, as treatment with the microtubule‐destabilizing drug Nocodazole interferes with ePCM foci formation. In contrast, chemical inhibition of the motor protein Eg5^KIF11^, which is essential for bipolar spindle formation, but not microtubule nucleation, results in the formation of a single ePCM focus in the center of the DNA mass, suggesting that Eg5^KIF11^ does not play a role in ePCM formation.

CEP192 (2537 amino acids), Pericentrin (PCNT, 3336 amino acids), and CDK5RAP2 (1893 amino acids) are large proteins with multiple domains that enable them to localize to the centrosome and interact with centrosomal proteins, PLK1 and γ‐TuRCs, which are essential for microtubule nucleation (Wieczorek et al. 2020; Zupa et al. 2021). CDK5RAP2 contains two key domains known for promoting various interactions: the N‐terminal CM1 domain, which is crucial for γ‐TuRC binding, and the C‐terminal CM2 domain, which facilitates both intramolecular interactions and binding to PCNT (Choi et al. 2010; Citron et al. 2018; Feng et al. 2017; Fong et al. 2008; Kim and Rhee 2014; Samejima et al. 2008; Wang et al. 2010; Zhang and Megraw 2007). Deletion of either domain disrupts ePCM foci and bipolar spindle formation, indicating that CDK5RAP2 links microtubule nucleation with ePCM foci formation to support bipolar spindle formation in cells lacking centrosomes (Watanabe et al. 2020). Several mechanisms involving CEP192 and PCNT in centrosome assembly have been described. CEP192 directly binds and recruits PLK1 to centrosomes, driving PCM expansion and γ‐TuRC recruitment during mitosis, a process known as centrosome maturation (Joukov et al. 2014; Meng et al. 2015). PCNT, phosphorylated by PLK1, promotes centrosome maturation and is recruited to centrosomes through direct binding with CEP57 (Lee and Rhee 2011; Watanabe et al. 2019). While the role of these mechanisms in ePCM formation remains undetermined, it is likely that multiple domains within CEP192 and PCNT, which support centrosomal localization and intermolecular interactions, are also crucial for bipolar spindle formation in acentrosomal cells.

Interestingly, some cancer‐derived cell lines that can divide without centrosomes do not exhibit ePCM foci, suggesting the presence of alternative pathways for acentrosomal spindle formation in a cancer‐specific context (Chinen et al. 2021; Watanabe et al. 2020). Chinen et al. (2020) proposed a crucial role for NuMA in centrosome‐independent spindle bipolarization. In normal cells with centrosomes, NuMA clusters astral microtubules near the centrosomes, aiding in spindle positioning and organization (Gaglio et al. 1995; Hueschen et al. 2017; Kisurina‐Evgenieva et al. 2004; Merdes et al. 2000; Merdes et al. 1996). Chinen and colleagues suggested a model for acentrosomal spindle formation in which dynein facilitates NuMA aggregation with microtubule asters during the onset of mitosis, leading to the formation of two poles. These poles are then separated via kinetochore‐microtubule attachment and the activity of the kinesin motor protein Eg5^KIF11^. The authors propose that while this pathway complements bipolar spindle assembly in conventional centrosomal cell division, it plays a predominant role in acentrosomal cells as a compensatory mechanism (Chinen et al. 2020).

## 7 Acentrosomal Cell Division Activates p53

**FIGURE 4:**
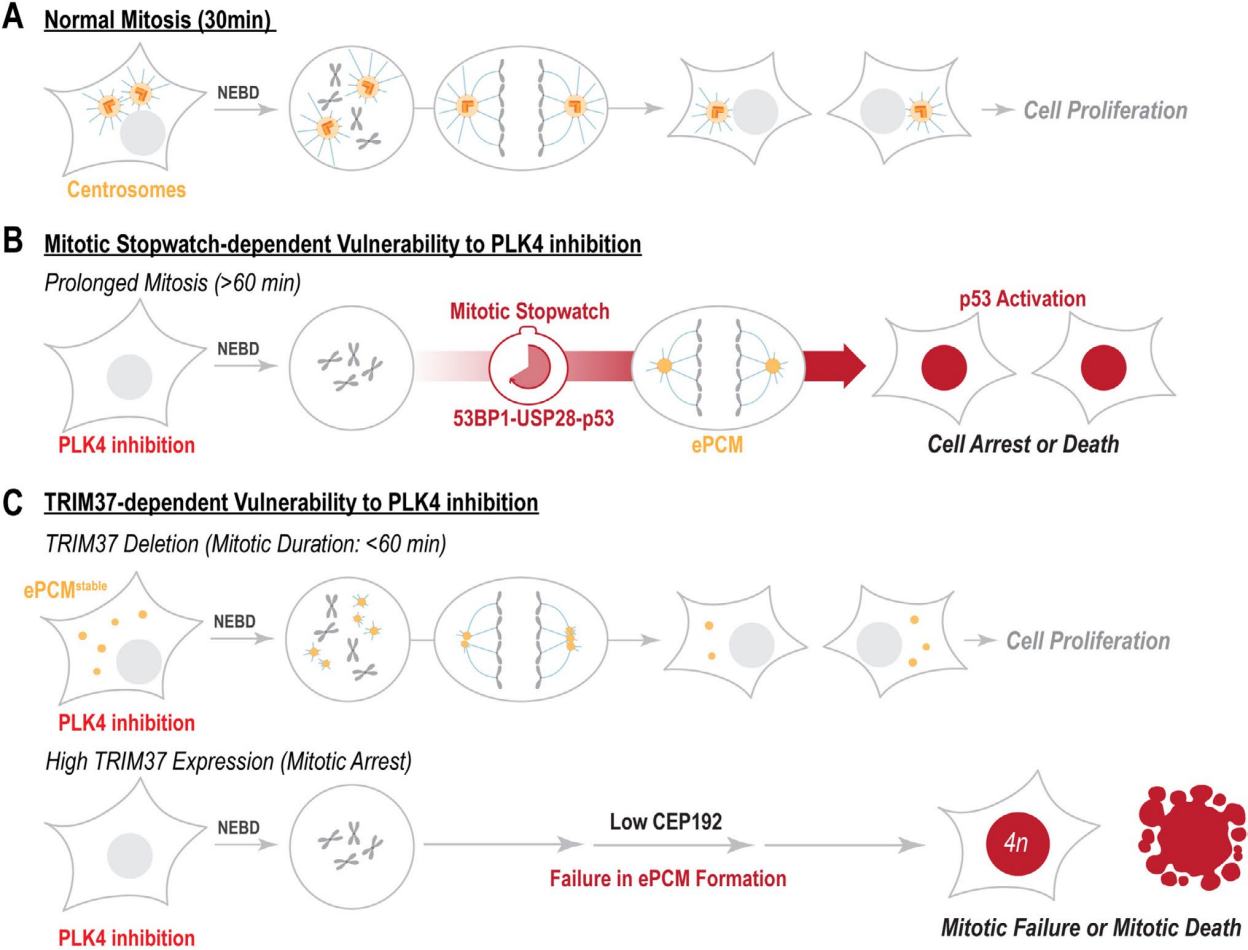
Cancer‐specific vulnerability to PLK4 inhibition. (A) Illustration of normal mitosis, typically completed within approximately 30 min. (B) In acentrosomal cells, mitosis is prolonged, activating a “mitotic stopwatch” that stabilizes the tumor suppressor p53 to halt cell proliferation. (C) Deletion of TRIM37 accelerates acentrosomal mitosis by promoting the formation of stable ectopic pericentriolar material (ePCM^stable^) foci, which functionally compensate for centrosomes. In contrast, TRIM37 amplification inhibits ePCM formation during mitosis, leading to mitotic failure or cell death.

Despite their ability to form bipolar spindles, non‐transformed human retinal pigment epithelial (hTERT RPE‐1) cells undergo p53‐dependent G1 arrest following centrosome depletion (Mikule et al. 2007). A similar arrest was observed in cells that experienced mitosis lasting longer than 90 min, even in the presence of centrosomes (Uetake and Sluder 2010), suggesting that the prolonged duration of mitosis in acentrosomal cells, rather than the absence of centrosomes alone, triggers p53 activation (Bazzi and Anderson 2014). The mitotic surveillance pathway, also known as the mitotic stopwatch, is now well‐established as the key mechanism that induces cell arrest or death after extended mitosis (Figure 4A,B) (Belal et al. 2024). This pathway appears to function across different cell types, as studies have shown that human embryonic stem cells (H1) and human mammary gland epithelial cells (MCF10A) exhibit a similar response to prolonged mitosis as hTERT RPE‐1 cells (Meitinger et al. 2024).

Three independent research groups conducting genome‐wide CRISPR‐Cas9 screens identified ubiquitin‐specific protease 28 (USP28) and p53‐binding protein 1 (53BP1) as core components of the mitotic stopwatch (Figure 4B) (Fong et al. 2016; Lambrus et al. 2016; Meitinger et al. 2016). During prolonged mitosis, 53BP1 was found to form a stopwatch complex with USP28 and p53 in a PLK1‐dependent manner (Meitinger et al. 2024). Once formed, the stable stopwatch complex persisted after mitosis and throughout the following interphase, where it stabilized p53, leading to the activation of cell cycle inhibitor p21 or pro‐apoptotic genes such as PUMA and BAX.

The mitotic stopwatch holds physiological relevance in the context of development and maintenance of healthy tissues (Belal et al. 2024). Developing embryos lacking centrioles extend their mitotic duration and activate the mitotic stopwatch at around E7 of development and arrest at around E9 during mid‐gestation (Bazzi and Anderson 2014; Grzonka and Bazzi 2024; Xiao et al. 2021). Prolonged mitosis driven by centrosome loss has detrimental effects on epidermal thickness, lung branching, and development of kidney and neural stem cells (Damen et al. 2021; Insolera et al. 2014; Langner et al. 2024; Xiao et al. 2021; Xie et al. 2021). Some of these defects were linked to mitotic stopwatch‐mediated activation of p53 (Damen et al. 2021; Grzonka and Bazzi 2024; Langner et al. 2024; Xiao et al. 2021). Mutations in centrosomal genes are one of the main causes of primary microcephaly (Phan and Holland 2021). Work by Phan and colleagues found that microcephaly‐associated mutations in the centrosomal genes CEP63 and CPAP cause a prolonged mitosis phenotype in neuronal progenitor cells in a mouse model for primary microcephaly. The “small brain” phenotype characteristic of primary microcephaly could be partially rescued by USP28 or 53BP1 deletion, suggesting that chronic activation of p53 contributes to this neurodevelopmental disorder (Phan et al. 2021). Considering that the mitotic stopwatch eliminates cells with mitotic defects, this pathway is likely in place to protect tissues from potentially dangerous cells with unstable genomes.

## 8 Cancer‐Specific Vulnerability to PLK4 Inhibition

Around 50% of cancers express wild‐type p53, suggesting that they may possess a functional mitotic stopwatch, which would increase their susceptibility to PLK4 inhibition (Figure 4A,B). Surprisingly, the mitotic stopwatch was active in only about 30% of the p53 wild‐type cancer cell lines tested, many originating from pediatric cancers (Meitinger et al. 2024). As anticipated, the presence of an active mitotic stopwatch correlated with increased sensitivity to anti‐mitotic drugs that extend mitosis, including the clinically relevant microtubule depolymerization inhibitor Taxol, the kinesin CENPE inhibitor GSK923295, and the PLK4 inhibitor Centrinone. Deletion of USP28 or 53BP1 reduced the sensitivity of the neuroblastoma cell line CHP134 to all three drugs, reinforcing that the sensitivity to anti‐mitotic drugs relies on mitotic stopwatch function.

Loss of stopwatch function was linked either directly to mutations in stopwatch complex genes or indirectly to mutations in genes that suppress p53 activity. Two of the analyzed cell lines carrying wild‐type p53 exhibited frameshift mutations and deletions that disrupted USP28 expression, supporting the hypothesis that the mitotic stopwatch pathway acts as a tumor suppressor. Two other cancer cell lines (HCT116, U2OS) harbored a truncation in Wip1^PPM1D^, leading to stabilization and hyperactivation of its phosphatase domain, which in turn inhibited p53 (Kleiblova et al. 2013). Chemical inhibition of Wip1^PPM1D^ partially restored mitotic stopwatch activity (Meitinger et al. 2024). These findings suggest that the status of the mitotic stopwatch in cancer cells could serve as a potential biomarker for targeted cancer therapies.

**FIGURE 5:**
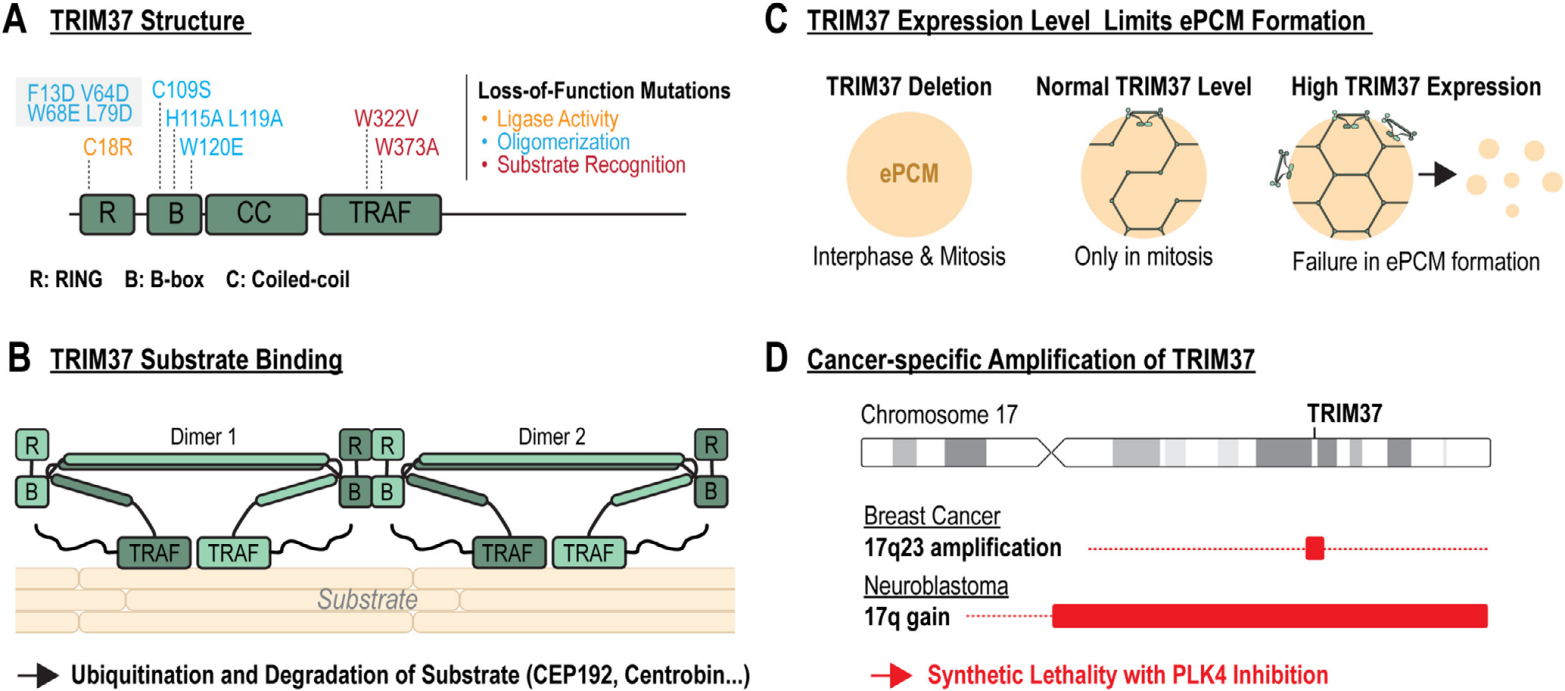
TRIM37 inhibits ePCM formation via its ubiquitin ligase activity. (A) Schematic representation of the TRIM37 protein structure with annotated mutations that disrupt specific functions. (B) Illustration of TRIM37 oligomerization, enabling it to bind substrates and promote their ubiquitylation and subsequent degradation via the proteasome. (C) Diagram showing how varying TRIM37 expression levels influence the formation of ectopic pericentriolar material (ePCM) foci. (D) Increased TRIM37 expression resulting from the amplification of chromosome 17q23 in breast cancer or the gain of chromosome 17q in neuroblastoma sensitizes these cancers to PLK4 inhibition.

The genetic screen that identified the mitotic stopwatch genes also revealed that knocking out the ubiquitin ligase TRIM37 allowed hTERT RPE‐1 cells to proliferate without centrosomes (Meitinger et al. 2016). The discovery of TRIM37 as a factor that confers cancer‐specific vulnerability started with the investigation of its loss‐of‐function phenotype. Interestingly, TRIM37 deletion did not disrupt the mitotic stopwatch function but shortened the mitotic duration of acentrosomal cells (Figure 4C). Consequently, the mitotic stopwatch was not activated in acentrosomal cells. In contrast, overexpression of TRIM37 leads to mitotic failure or death in acentrosomal cells (Meitinger et al. 2020; Yeow et al. 2020). TRIM37 is an E3 ubiquitin ligase featuring an N‐terminal RBCC domain (composed of a RING domain, B‐box domain, and coiled‐coil region), a TRAF domain, and an unstructured C‐terminal tail (Figure 5A). Loss‐of‐function mutations in TRIM37 are linked to Mulibrey Nanism, a tumor‐prone disorder that affects multiple tissues, including muscle, liver, brain, and eye (Avela et al. 2000; Karlberg et al. 2009).

How does TRIM37 deletion suppress the prolonged mitosis phenotype in acentrosomal cells? Several studies suggest that TRIM37 deletion results in the accumulation of centrosomal proteins, particularly CEP192 and Centrobin, indicating that TRIM37 may ubiquitinate these proteins to target them for proteasomal degradation (Balestra et al. 2021; Balestra et al. 2013; Meitinger et al. 2021; Meitinger et al. 2020; Yeow et al. 2020). Consequently, TRIM37 deletion could lead to an accumulation of centrosomal proteins that promote the formation of a bipolar spindle in acentrosomal cells. In fact, in PLK4‐inhibited acentrosomal cells, TRIM37 deletion led to the formation of stable ePCM (ePCM^Stable^) foci, containing PCM and centriole‐associated proteins such as CEP192, CEP152, CDK5RAP2, PCNT, and HsSAS‐6 (Figure 4C) (Meitinger et al. 2016). The absence of the essential centriolar gene CPAP confirmed that these structures were not dependent on traditional centrosomes. Importantly, these ectopic structures recruited γ‐TuRCs, which are critical for microtubule nucleation and bipolar spindle formation (Meitinger et al. 2016). A key component of ePCM^Stable^ is CEP192, which is enriched in TRIM37‐deleted cells. In contrast to the previously mentioned ePCM foci, ePCM^Stable^ foci are also present in interphase cells and are independent of microtubule nucleation and PLK1 activity. Taken together, these findings suggest that TRIM37 plays a role in targeting centrosomal proteins for degradation, thereby preventing the ectopic formation of centrosome‐like structures.

The function of TRIM37 has been shown to depend on its ubiquitin ligase activity, reinforcing the suggestion that TRIM37‐mediated ubiquitination targets substrates for proteasomal degradation (Figure 5A) (Meitinger et al. 2020). The C18R ligase‐dead mutation also led to increased TRIM37 protein abundance, suggesting that TRIM37 regulates its own degradation. Additionally, the mutant accumulated at the centrosome, indicating that the ligase‐dead variant may be trapped by centrosomal substrates. Furthermore, it was shown that the TRAF domain mutant W373A interfered with centrosome localization, highlighting the role of the TRAF domain in substrate recognition. Two recent studies gave insight into how TRIM37 recognizes and binds to the substrate (Bellaart et al. 2024; Yeow et al. 2024). The authors demonstrated that the RING domain is the major driver for TRIM37 oligomerization, which is required for substrate binding (Figure 5A,B). In contrast, the B‐box interface was necessary for substrate recognition and may play a role in higher‐order assemblies at the substrate. Targeted mutations in both domains interfered with TRIM37 recruitment to centrosomes. These findings suggest that TRIM37 employs a similar substrate targeting mechanism to TRIM5, which detects and initiates the degradation of retroviral nucleocapsid shells in the cytoplasm (Figure 5B,C) (Ganser‐Pornillos and Pornillos 2019).

The TRIM37 gene is located at the 17q22‐17q23 region, which is amplified in numerous tumor types, particularly in breast cancer and neuroblastoma (Figure 5D) (Andersen et al. 2002; Ho et al. 2018; Parssinen et al. 2007). Importantly, cancers with amplified TRIM37 became increasingly vulnerable to PLK4 inhibition, which led to mitotic failure or death (Figures 4C and 5D) (Meitinger et al. 2020; Yeow et al. 2020). The sensitivity to PLK4 inhibition correlated directly with TRIM37 protein expression level (Meitinger et al. 2020). Overexpression of TRIM37 sensitized hTERT RPE‐1 cells, which subsequently failed to form ePCM foci (Figures 4C and 5C). Increased degradation of CEP192, which is required for ePCM formation, was found to cause mitotic failure in PLK4‐inhibited cells. In line with this observation, partial depletion of CEP192 phenocopied the effect of TRIM37 overexpression. These studies highlight the potential of PLK4 inhibition in treating cancers with TRIM37 amplifications such as breast cancer and neuroblastoma.

## 9 Conclusion

PLK4 is the master regulator of centriole duplication. Significant progress has been made in our understanding of how centrioles duplicate and how centrosomes mature to drive accurate chromosome segregation. A synthetic lethal relationship between PLK4 inhibition and TRIM37 overexpression in cancers with amplifications or gains of the chromosomal region 17q23, commonly observed in breast cancer and neuroblastoma, has brought PLK4 inhibitors into the spotlight as potential therapeutics. To employ this synthetic lethality in clinical trials, highly specific PLK4 inhibitors are required. While highly specific, the PLK4 inhibitor Centrinone is not suitable for clinical studies due to poor pharmacokinetics. Also, the pan‐kinase inhibitor CFI400945 may not exhibit synthetic lethality with TRIM37 amplification due to its off‐target effects (Mason et al. 2014; Oegema et al. 2018; Wong et al. 2015). A recent report announced the successful development of a highly specific PLK4 inhibitor RP‐1664 suitable for clinical trials (Therapeutics 2023). Like Centrinone, RP‐1664 exhibited a synthetic lethality with TRIM37 amplification. Furthermore, the compound induced tumor regression in patient‐derived xenograft models of breast (triple negative and ER positive) and non‐small cell lung cancers as well as in cell line‐derived xenograft models of the neuroblastoma cell lines CHP134 and IMR32 (Therapeutics 2023). In 2024, the first clinical trial (Phase 1) for RP‐1664 was announced. While the clinical potential of PLK4 inhibitors is currently under investigation, the discovery of the synthetic lethal relationship with TRIM37 amplification has provided a promising therapeutic avenue.

## Author Contributions

All authors contributed to the conceptualization and writing of the manuscript.

## Conflicts of Interest

The authors declare no conflicts of interest.

## Data Availability

Data sharing not applicable to this article as no datasets were generated or analysed during the current study.
